# *Osa-miR7695* enhances transcriptional priming in defense responses against the rice blast fungus

**DOI:** 10.1186/s12870-019-2156-5

**Published:** 2019-12-18

**Authors:** Ferran Sánchez-Sanuy, Cristina Peris-Peris, Shiho Tomiyama, Kazunori Okada, Yue-Ie Hsing, Blanca San Segundo, Sonia Campo

**Affiliations:** 1 Centre for Research in Agricultural Genomics (CRAG) CSIC-IRTA-UAB-UB, Campus Universitat Autònoma de Barcelona (UAB), Bellaterra (Cerdanyola del Vallés), Barcelona, Spain; 2 Biotechnology Research Center, The University of Tokyo, Tokyo, Japan; 3 Institute of Plant and Microrbial Biology, Academia Sinica, Taipei, Taiwan; 4 Consejo Superior de Investigaciones Científicas (CSIC), Barcelona, Spain

**Keywords:** Blast, Defense, Iron, *Magnaporthe oryzae*, microRNA, miR7695, *Oryza sativa*, Phytoalexins, Rice, Transcriptomics

## Abstract

**Background:**

MicroRNAs (miRNAs) are small non-coding RNAs that regulate gene expression at the post-transcriptional level in eukaryotes. In rice, *MIR7695* expression is regulated by infection with the rice blast fungus *Magnaporthe oryzae* with subsequent down-regulation of an alternatively spliced transcript of *natural resistance-associated macrophage protein 6* (*OsNramp6*). NRAMP6 functions as an iron transporter in rice.

**Results:**

Rice plants grown under high iron supply showed blast resistance, which supports that iron is a factor in controlling blast resistance. During pathogen infection, iron accumulated in the vicinity of *M. oryzae* appressoria, the sites of pathogen entry, and in cells surrounding infected regions of the rice leaf. Activation-tagged *MIR7695* rice plants (*MIR7695*-Ac) exhibited enhanced iron accumulation and resistance to *M. oryzae* infection. RNA-seq analysis revealed that blast resistance in *MIR7695-*Ac plants was associated with strong induction of defense-related genes, including pathogenesis-related and diterpenoid biosynthetic genes. Levels of phytoalexins during pathogen infection were higher in *MIR7695-*Ac than wild-type plants. Early phytoalexin biosynthetic genes, *OsCPS2* and *OsCPS4*, were also highly upregulated in wild-type rice plants grown under high iron supply.

**Conclusions:**

Our data support a positive role of miR7695 in regulating rice immunity that further underpin links between defense and iron signaling in rice. These findings provides a basis to better understand regulatory mechanisms involved in rice immunity in which miR7695 participates which has a great potential for the development of strategies to improve blast resistance in rice.

## Background

Plants have a sophisticated innate immune system for protection against pathogen infection [1, 2]. The activation of plant defense responses against pathogens occurs via the recognition of conserved pathogen-associated molecular patterns (PAMPs; previously known as elicitors) by host pattern-recognition receptors (PRR) which, in turn, triggers a signaling cascade leading to the activation of defense-related responses. Pathogen-induced defense responses include the production of reactive oxygen species (ROS), the activation of protein phosphorylation/dephosphorylation cascades, and the production of pathogenesis-related (PR) proteins, among others [3, 4]. Successful pathogens, however, have developed countermeasures to suppress this basal defense in certain plant species and promote disease by delivering effectors into the host. Plants have also evolved Resistance (R) genes that recognize microbial effectors to activate a much stronger immune response, the so called effector-triggered immunity [5]. PTI and ETI have long been considered protein-based mechanisms. However, increasing evidence supports that microRNAs (miRNAs) are also important players in both PTI and ETI [6–11].

MiRNAs are small noncoding RNAs that modulate gene expression in eukaryotes by triggering sequence-specific cleavage or translational repression of target genes [12]. Plant miRNAs play a crucial role in the control of developmental processes and adaptation to environmental stresses, both abiotic and biotic stresses [13–16]. Although numerous miRNAs have been reported to be regulated during pathogen infection, the biological role of most of them remains unknown. Furthermore, these studies have been conducted mainly in the model dicotyledonous plant *Arabidopsis thaliana* during interaction with the bacterial pathogen *Pseudomonas syringae*. Further experimental validation is required to better understand the regulatory roles of miRNAs in plant immunity.

In the past few years, studies have demonstrated that miRNAs act as regulators of nutrient homeostasis in plants by modulating the expression of genes involved in nutrient homeostasis [17]. It has been shown that miR399 and miR395 play a fundamental role in phosphate and sulfur homeostasis in plants [18, 19]. Plant miRNAs controlling nutrient homeostasis may also be important factors in controlling disease resistance. Unfortunately, miRNA-mediated mechanisms involved in disease resistance and nutrient homeostasis have been studied separately.

Iron (Fe) is an essential microelement for plant growth required for essential redox reactions in metabolism. Fe is also required for photosynthesis and maintenance of chloroplast function [20]. However, excess Fe generates reactive oxygen species (ROS), which might cause oxidative damage to macromolecules (e.g. nucleic acids, lipids, proteins) and cellular structures [21–25]. During pathogen infection, Fe homeostasis must be carefully regulated as the host and pathogen compete for the available Fe. The pathogen must acquire this vital element from host tissues, whereas the host plant can deprive the invader of Fe as a defensive strategy. Mechanisms for maintaining Fe homeostasis need to be highly dynamic in the host plant to allow normal plant growth. Although distinct miRNAs have been shown to be responsive to Fe stress [26–30], how such alterations will affect Fe homeostasis and disease resistance remain to be determined.

Rice is one of the most important cereal crops in the world and the model plant for genomics research of monocotyledonous [31, 32]. Rice production is severely affected by blast disease caused by the fungal pathogen *Magnaporthe oryzae* [33]. miRNAs controlling traits of agronomic importance (e.g., tiller growth, early flowering, grain production) [34–36] and tolerance to abiotic stress (drought, salinity and cold stress) [37–39] have been described in rice. Evidence also supports variations in the accumulation of rice miRNAs during *M. oryzae* infection or treatment with *M. oryzae* elicitors [40–43], but the biological function of only a few of these pathogen-regulated miRNAs has been demonstrated. They include both positive regulators (miR7695, miR160, miR398, and polycistronic miR166k-166 h) and negative regulators (miR164a, miR169 and miR319) of defense responses against the rice blast fungus *M. oryzae* [41, 42, 44–47]. Hence, to obtain a comprehensive understanding of the regulatory functions of miRNAs in the rice response to *M. oryzae* infection, intense experimental validation of miRNA functioning is mandatory.

We previously reported that the rice miR*7695* is involved in blast resistance [41]. This particular miRNA targets an alternatively spliced transcript of *OsNramp6* (*natural resistance-associated macrophage pathogen 6*), in particular the shortest transcript variant (*OsNramp6.8),* encoding an Fe and manganese transporter, the NRAMP6 protein [48]. Here we show that Fe accumulates at the sites of pathogen penetration (appressoria) and cells surrounding the infection sites in *M.oryzae*-infected rice leaves. *MIR7695* activation results in blast resistance which was associated with local iron accumulation at the infection sites and superinduction of *PR* and diterpenoid phytoalexin biosynthesis genes. Consequently, *MIR7695* activation plants accumulated major rice phytoalexins in their leaves. These results provide new insights into the role of miR7695 in regulating immune responses and Fe signaling pathways in the rice–*M. oryzae* interaction.

## Results

### *M. oryzae* infection alters Fe distribution in rice leaves

**Fig. 1:**
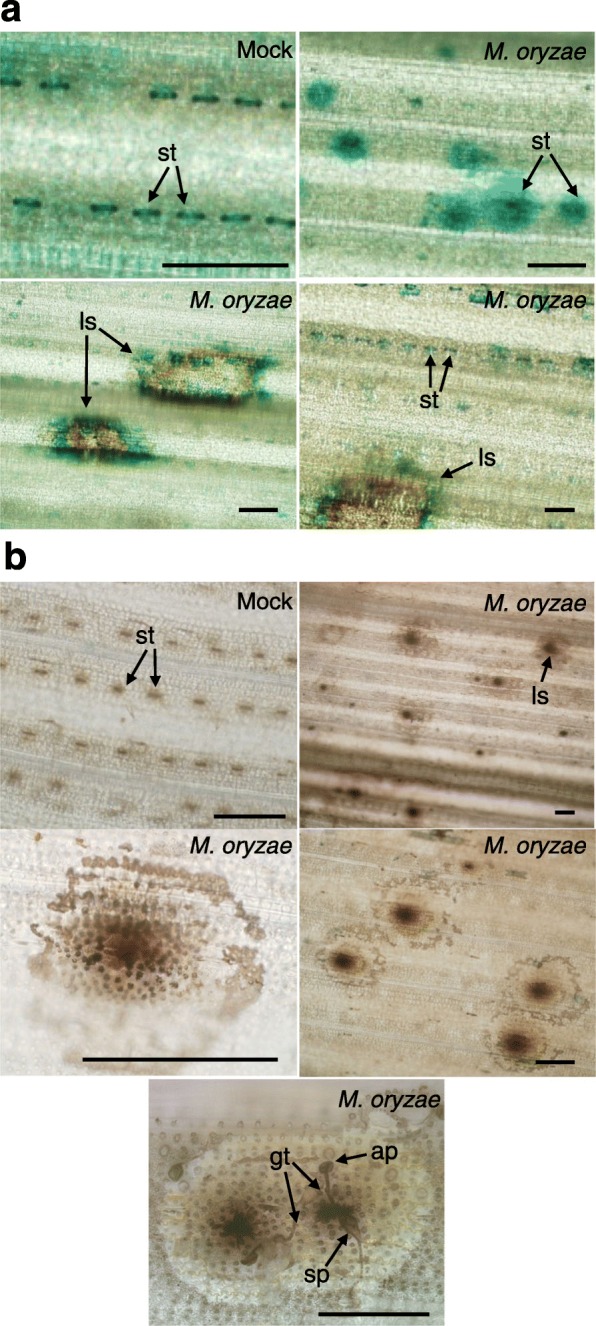
Histochemical detection of iron (Fe) in *M. oryzae*-infected rice (*O. sativa* cv. Nipponbare, *japonica*) leaves. Wild-type plants at the three-leaf stage were sprayed with a *M. oryzae* spore suspension or mock inoculated. At 24–48 h post-inoculation (hpi), the third leaf of each plant was stained with Perls (**a**) or Perls/DAB (**b**) (blue and black precipitates, respectively). Scale bar: 100 μm. ap, appressorium; gt, germ tube; ls, lesion; st, stomata; sp, spore

In this work, we investigated the cellular distribution of Fe during *M. oryzae* infection by using the Perls staining. Perls reagent (potassium ferrocyanide) reacts with Fe^3+^ to form an insoluble pigment, or Prussian blue. Without pathogen infection, Perls staining revealed that Fe preferentially accumulated at stomata (Fig. 1a upper left panel). Upon pathogen challenge, iron staining showed a less uniform, but more widespread distribution in the stomatal areas pointing to a possible pathogen-induced iron mobilization (Fig. 1a upper right panel). Of interest, Perls staining revealed iron accumulation forming halo areas around the infection sites (Fig. 1a, lower panels).

To increase the sensitivity and definition of Fe detection in rice leaves, we used intensified Perls staining with DAB/H_2_O_2_. This method takes advantage of the redox activity of the Prussian blue reagent. As previously observed by Perls staining, after Perls/DAB staining, strong black precipitates appeared at stomatal areas in mock-inoculated leaves which showed a diffuse staining upon *M. oryzae* infection (Fig. 1b, upper panels). In those regions, Fe-stained granules were often visible (Fig. 1b, middle left panel). As well, in these regions, Fe accumulated with different intensities, with strong black precipitates in the center, surrounded by weaker and unevenly distributed halos of black precipitate (Fig. 1b, middle right panel). Higher magnification of these regions showed germinating spores and germ tubes forming appressoria, the sites where pathogen entry occurs (Fig. 1b, lower panel). Fe was weakly stained further away from the penetration site. Hence, histochemical analysis of Fe accumulation established that Fe accumulates at the sites of attempted penetration by the fungus (appressoria) as well as in cells in close proximity to the infection site, supporting that Fe distribution might be important for blast resistance.

### Resistance to infection by the rice blast fungus *M. oryzae* in mutant plants with *MIR7695* activation

**Fig. 2:**
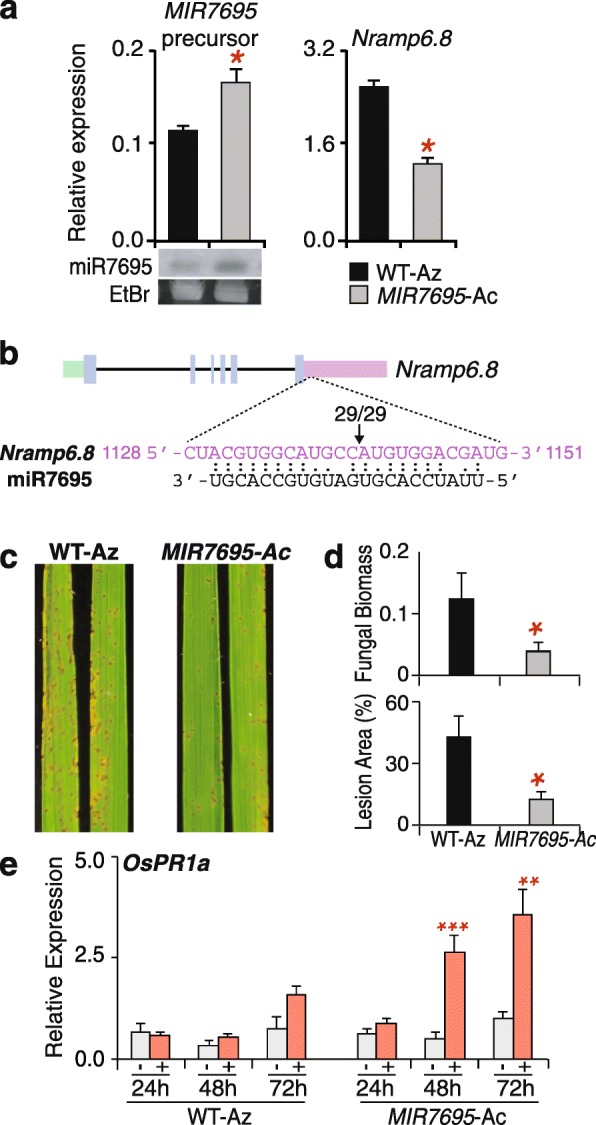
Resistance of *MIR7695-Ac* mutant plants to *M. oryzae* infection. **a** RT-qPCR analysis of *MIR7695* precursor transcripts (left panel) and miR7695 target (*Nramp6.8,* Os01g0503400.8) in homozygous mutant (*MIR7695*-Ac) and WT (segregated azygous, WT-Az) plants. Data are mean ± SE (*n* = 3) (Student *t* test, *p < 0.05). Lower panel: northern blot analysis of mature miR7695 using the miR7695.3-3p sequence as the hybridization probe (Additional file 2: Table S1). As a loading control, the RNA blot was stained with ethidium bromide (EtBr) (**b**) Experimental validation of miR7695-mediated cleavage of *OsNramp6.8* transcripts by 5′-RLM-RACE. Schematic representation of the *OsNramp6.8* (upper panel), showing the coding sequence (blue), 5’UTR (green), and 3’UTR (pink). Boxes, exons; lines, introns. Gene-specific primers were used for 5′-RACE and the resulting PCR products were sequenced. The identified cleavage site is indicated by an arrow and the number above indicate the detected cleavage site of independent clones. **c** Leaves of 3-week-old plants were sprayed with a *M. oryzae* spore suspension. The second leaf was photographed at 7 days post-inoculation. **d** Percentage of leaf area affected by blast lesions (upper panel). Relative fungal biomass (lower panel) was determined by qPCR as the ratio of *M. oryzae* 28S ribosomal DNA to the rice *Ubiquitin1* gene (primers in Additional file 2: Table S1). Data are mean ± SE (*n* = 7) from 1 experiment (Student *t* test, *p < 0.05). Four independent infection assays were performed with similar results. **e** RT-qPCR analysis of *OsPR1a* transcripts at different times after inoculation with *M. oryzae* spores. Blast infection was carried out as in (**c**). Data are mean ± SE (*n* = 3, each biological replicate is a pool of 3 individual leaves) (Student *t* test, **p < 0.01 ***p < 0.001; infected vs non-infected). Mock inoculated (control) plants; +, *M. oryzae*-infected plants.

We searched publicly available rice mutant collections for mutants with affected *MIR7695* expression. Because of the small size of *MIR* genes, identifying mutant alleles for miRNAs in insertional mutant collections is unlikely. A T-DNA tagged line (M0107013) was identified in the Taiwan Rice Insertion Mutants (TRIM) Database [49]; http://trim.sinica.edu.tw) in which the T-DNA was inserted upstream of the *MIR7695* locus (Additional file 1: Figure S1a, left panel). TRIM was designed for gene knockout and activation tagging in the Tainung67 (*japonica*) background. Thus, the presence of an octamer of the *cauliflower mosaic virus 35S* (*CaMV35*) transcriptional enhancer next to the left border of the T-DNA can activate the expression of genes located up to 30 Kb from the integration site [50, 51] (Additional file 1: Figure S1a, left panel). Homozygous and azygous plants were identified by PCR genotyping (Additional file 1: Figure S1a, right panel, primers are in Additional file 2: Table S1). Quantitative PCR (qPCR) revealed that *MIR7695-*Ac plants had a single copy of T-DNA inserted in its genome (Additional file 3: Table S2) Importantly, the accumulation of miR7695 precursor and mature sequences was higher in homozygous mutant plants with *MIR7695* activation tagging (hereafter *MIR7695-*Ac) than wild-type azygous (WT-Az) plants as revealed by RT-qPCR and small-RNA northern blot analyses, respectively (Fig. 2a, left panel). Consistent with upregulated *MIR7695*, the accumulation of miR7695 target transcripts (*OsNramp6.8*) was decreased in leaves with *MIR7695-*Ac (Fig. 2a, right panel).

We previously reported that the recognition site of miR7695 locates in the 3′ UTR region of *OsNramp6.8* transcripts. In this study, we further investigated whether *OsNramp6.8* gene is a real target gene for miR7695 by performing RNA ligase-mediated 5′ RACE (5′-RLM-RACE). Sequencing of the 5′ -RACE PCR products identified cleavage fragments at the expected site of *OsNramp6.8* transcripts, thus, supporting that *OsNramp6.8* transcripts are cleaved by miR7695 (Fig. 2b). These observations demonstrated that M0107013 is an activation mutant for *MIR7695* (*MIR7695-*Ac plants) and that miR7695 cleaves *OsNramp6.8* transcripts. *MIR7695-*Ac plants were slightly shorter and contained less chlorophyll than did WT-Az plants, but these differences were not statistically significant (Additional file 1: Figure S1b and c).

Infection experiments were performed to assess the effect of *MIR7695* activation on disease resistance. WT-Az and *MIR7695-*Ac plants were spray-inoculated with *M. oryzae* spores. On visual inspection, *MIR7695*-Ac plants were more resistant to *M. oryzae* infection than were WT plants (Fig. 2c). Blast resistance was confirmed by quantifying the lesion area and the relative amount of fungal DNA in infected leaves (Fig. 2d). Resistance of *MIR7695-*Ac plants to *M. oryzae* infection was also observed by local inoculation of detached rice leaves (Additional file 4: Figure S2).

The induction of *PR1* expression is a widely used indicator of defense activation in response to pathogen infection in plants, including infection by *M. oryzae* in rice [52]. As expected, *PR1a* was induced in WT-Az plants during *M. oryzae* infection (Fig. 2e). However, *PR1a* was induced at a much higher level in fungal-infected *MIR7695-*Ac than WT-Az plants (Fig. 2e), which is consistent with the phenotype of blast resistance observed in *MIR7695-*Ac plants. The observed phenotype of blast resistance in *MIR7695-*Ac plants also agreed with resistance to *M. oryzae* infection in miR7695-overexpressing lines and *Osnramp6* mutant plants [41, 48].

**Fig. 3:**
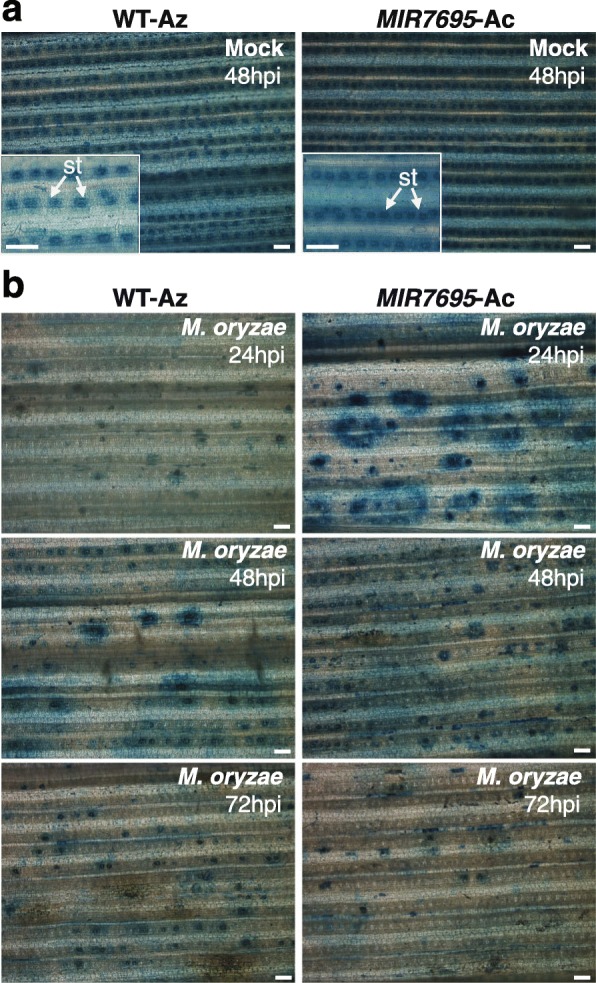
Histochemical detection of iron (Fe) in wild-type (*O. sativa* cv. Tainung 67, *japonica*) and *MIR7695*-Ac mutant plants during *M. oryzae* infection. Wild-type and *MIR7695-*Ac mutant plants at the three-leaf stage were (**a**) mock-inoculated or (**b**) inoculated with *M. oryzae* spores. At 24, 48, 72 h post-inoculation (hpi), the third leaf of each plant was stained with Perls. Iron is detected as blue precipitates. Representative images of one experiment are shown (*n* = 4). Three independent infection assays were performed with similar results. Scale bar: 100 μm. st, stomata

As previously mentioned, without pathogen infection, iron accumulated in the stomata of leaves from wild-type rice plants whereas *M. oryzae* infection induced iron mobilization to the infection sites in wild-type plants (see Fig. 1**)**. In this work, we determined the accumulation of iron at different time points after inoculation with *M. oryzae* spores in wild-type and *MIR7695*-Ac plants. As it was observed in wild-type plants, iron was detected in stomata of *MIR7695*-Ac leaves in non-infected plants (Fig. 3a). This analysis also revealed a stronger iron accumulation at the infection sites in the *MIR7695*-Ac plants compared to the WT-Az at 24 hpi (Fig. 3b, upper panels). Moreover, a general decrease on the iron content occurred at later time points (48 hpi, 72 hpi) in both wild-type and *MIR7695*-Ac plants.

### Transcript profiling of *MIR7695-*ac mutant plants

**Fig. 4:**
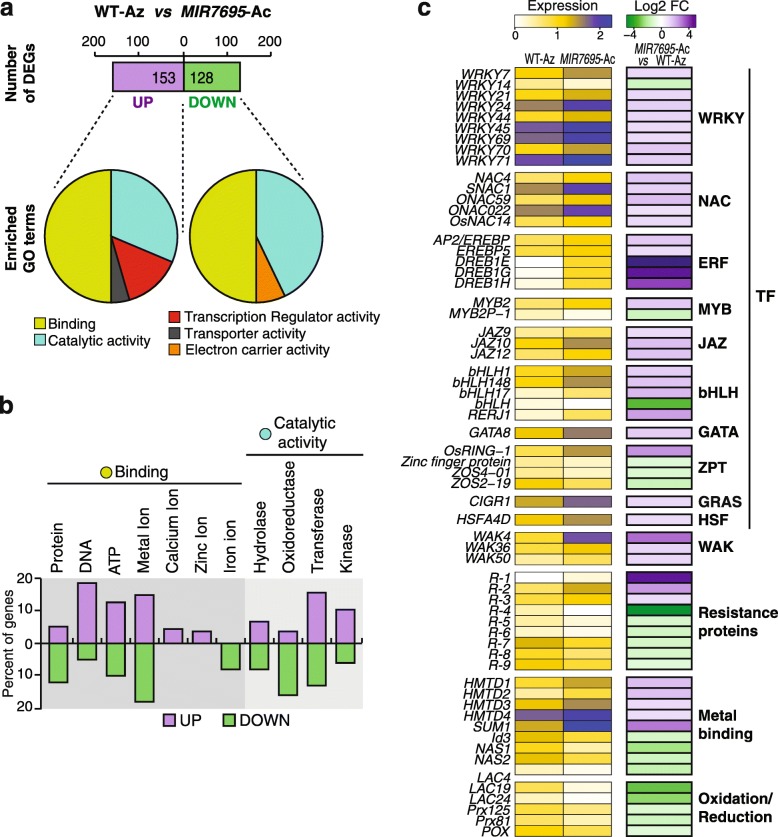
Differentially expressed genes (DEGs) in *MIR7695*-Ac mutant plants relative to WT-Az plants by RNA-seq analysis. Leaves of three-week-old plants were used (**a**) Number of DEGs and Gene Ontology (GO) analysis of DEG function. Up-regulated genes (log2 fold change [FC] ≥ 1; purple) and down-regulated genes (log2FC ≤ − 1; green) genes (*p* < 0.05, false discovery rate [FDR] < 0.05, *n* = 2). Pie charts represent the five general GO terms enriched in up- and downregulated DEGs. **b** Enriched terms in the “binding” and “catalytic activity” categories. **c** Heatmaps showing RNAseq expression level (left panel; log10 [FPKM+ 1]) and FC (right panel; log2FC) of DEGs. Gene expression is represented from pale yellow (less expressed) to blue (more expressed). Upregulated (log2FC ≥ 1; purple) and downregulated (log2FC ≤ − 1; green) DEGs. Data are means (*n* = 2). The full gene names and ID list are in Additional file 8: Table S5

To investigate the molecular mechanisms underlying blast resistance in *MIR7695* plants, we used RNA-seq analysis. Initially, we examined the impact of *MIR7695* activation on the rice transcriptome by comparing the transcript profiles of mock-inoculated *MIR7695*-Ac and WT-Az plants. We identified 281 differentially expressed genes (DEGs; 153 upregulated and 128 downregulated) (Fig. 4a; Additional file 5: Figure S3a). Additional file 6: Table S3 lists the DEGs in *MIR7695*-Ac plants. Singular enrichment analysis (SEA) of molecular function by using AgriGO revealed gene ontology (GO) annotations in the “binding” and “catalytic activity” categories, which were over-represented for both upregulated and downregulated DEGs (Fig. 4a; Additional file 7: Table S4). Genes in the categories “transcription regulator activity” and “transporter activity” were specifically enriched in the upregulated DEGs, whereas genes in the “electron carrier activity” category were enriched in downregulated DEGs (Fig. 4a). The binding category comprised genes related to “calcium ion binding” and “zinc ion binding” (upregulated only in *MIR7695-*Ac plants) and “iron ion binding” genes (downregulated only in *MIR7695-*Ac plants) (Fig. 4b).

The expression of a vast array of transcription factors (TFs) belonging to different TF families was regulated in mock-inoculated *MIR7695*-Ac plants (most of them being upregulated) (Fig. 4c; Additional file 8: Table S5). They included TFs with a demonstrated role in the rice defense response to blast infection), such as *OsWRKY45* and *OsNAC4* [53]. These TFs function as positive regulators of the rice response to *M. oryzae* infection [54, 55] and are both upregulated in *MIR7695-Ac* plants (Fig. 4c). Other TFs that are activated in *MIR7695* plants are known to mediate defense hormone signaling, such as ethylene response factor 5 (OsEREBP5), APETALA2/ethylene-responsive element binding protein (AP2/EREBP), several jasmonate ZIM-domain (JAZ) TFs, and *RERJ1* (a jasmonic acid-dependent stress inductive bHLH transcription factor) [56] (Fig. 4c). Genes encoding several wall-associated kinase (WAK) receptors and disease resistance (R) proteins were also upregulated in *MIR7695*-Ac (Fig. 4c). Upregulated genes in *MIR7695-Ac* plants also included several heavy metal transporter/metal detoxification (*HMTD*) protein genes and siroheme uroporphyrinogen methyltransferase1 (*SUM1*), encoding enzymes responsible for the synthesis of the Fe-containing cofactor of enzymes (Fig. 4c).

Genes that were downregulated in mock-inoculated *MIR7695-Ac* plants included those involved in the synthesis of nicotinamine (NA), a chelator of metals and the precursor of phytosiderophores (components for Fe acquisition) [57]: *OsNAS1* and *OsNAS2*, encoding nicotinamine synthases (Fig. 4c**).** Other downregulated genes are involved in oxidation-reduction processes, such as laccases (*OsLAC4, OsLAC19, OsLAC24*) and peroxidases (*Prx81, Prx125*) (Fig. 4c).

RT-qPCR was used to validate RNA-seq findings. RT-qPCR results obtained for selected genes were highly concordant with RNA-seq results for both upregulated genes (*OsWRKY45, OsWRKY71, OsNAC4, OsDREB1G, OsDREB1E*, *OsRERJ1*) and downregulated genes (*OsLAC19* and *OsNAS1*) (Additional file 9: Figure S4).

Together, these observations suggest that without pathogen infection, *MIR7695* activation led to altered expression of genes involved in 1) transcriptional regulation, 2) disease resistance, 3) metal binding and transport, and 4) oxidation-reduction mechanisms. Transcriptional changes caused by *MIR7695* activation might well contribute to the resistance response of these plants to pathogen infection.

### Enhanced defense responses to *M. oryzae* infection in *MIR7695*-ac plants

**Fig. 5:**
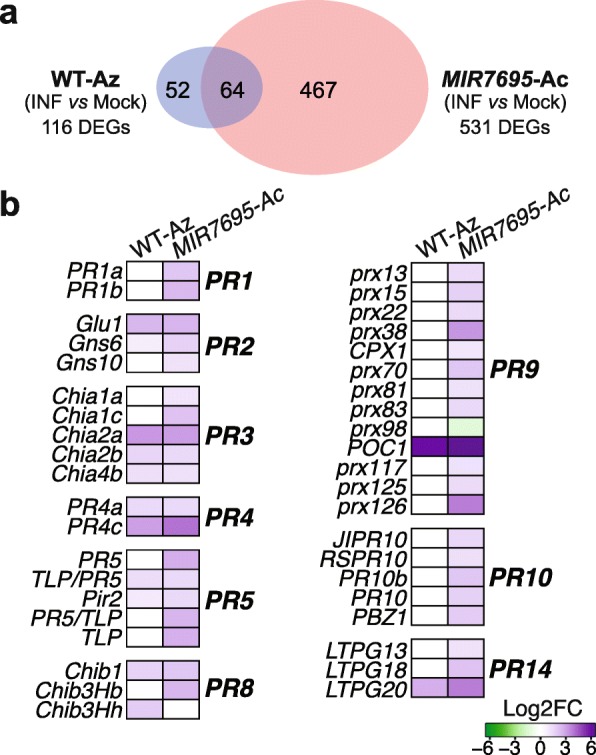
Comparison of DEGs in *MIR7695*-Ac and WT plants after challenge with *M. oryzae*. Leaves of 3-week-old rice plants (WT-Az and *MIR7695*-Ac) were mock-inoculated or sprayed with a suspension of *M. oryzae* spores, and collected at 48 hpi for RNA extraction and RNA-seq. Upregulated (log2FC ≥ 1) and downregulated (log2FC ≤ 1) genes by *M. oryzae* infection (*p* < 0.05, FDR < 0.05). **a** Venn diagram of the overlap between fungal-responsive genes of each genotype. **b** Comparison of the expression pattern of defense-related genes with *M. oryzae* infection. Up- (purple) and downregulated (green) DEGs. For a full list of gene IDs, see Additional file 12: Table S8

Pathogen-induced alterations in the transcriptome of *MIR7695*-Ac plants were identified and compared to those of fungal-infected WT-Az plants. The number of genes with expression affected by *M. oryzae* infection at 48 h post-infection (hpi) was 4.5 times higher in *MIR7695*-Ac than WT-Az plants (531 and 116, respectively) (Fig. 5a; Additional file 5: Figure S3bc). This observation already indicated stronger transcriptional regulation in the mutant plants. DEGs for WT-Az and *MIR7695*-Ac plants are listed in Additional files 10 and 11 (Tables S6 and S7), respectively. Of note, genes typically associated with the plant response to pathogen attack, such as *PR* genes, were induced in *MIR7695-Ac* but not in WT-Az plants at 48hpi (Fig. 5b; Additional file 12: Table S8). They included *PR1*, *β-1,3-glucanase* (*PR2*), *chitinase* (*PR3*, *PR4*, *PR8*), *thaumatin* (*PR5*), *peroxidase* (*PR9*), *PBZ1* and other *Bet v1* homologues (*PR10*), and *lipid transfer protein* (*LTP*; *PR14*). The antimicrobial activity of many of these PR proteins has been demonstrated (e.g., PR1, chitinases, β-1,3-glucanases, PR4, thaumatin, LTPs) [58].

**Fig. 6:**
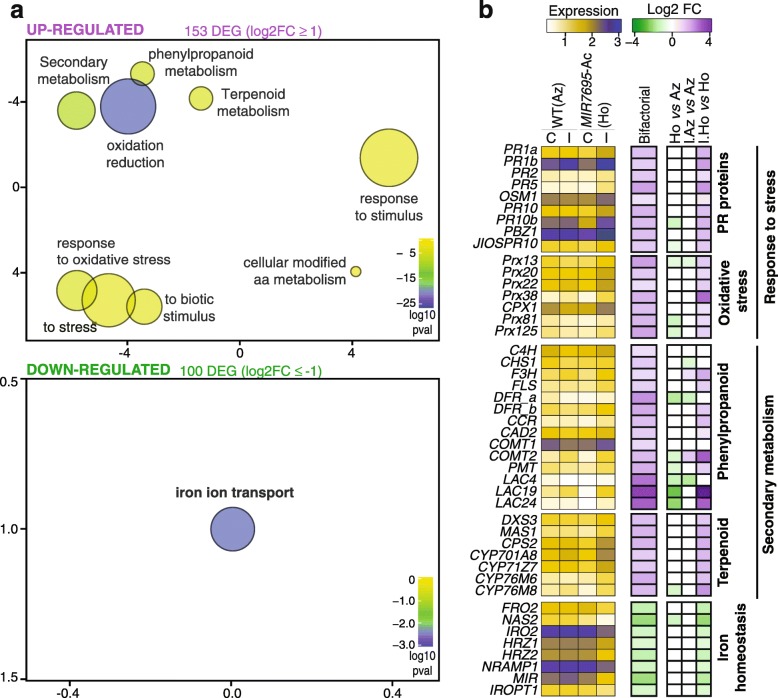
Biological processes altered in *MIR7695*-Ac mutant plants relative to WT-Az plants with *M. oryzae* infection. Same plant material as in Fig. 4. RNA-seq data underwent bifactorial analysis (upregulated, log2FC ≥ 1; downregulated, log2FC ≤ 1; *p* < 0.05, FDR < 0.05). **a** GO analysis of DEG function in *MIR7695*-Ac plants with blast infection (upper and lower panels show upregulated and downregulated DEGs, respectively). The top GO terms enriched in *MIR7695*-Ac vs WT-Az DEGs were represented by using REVIGO after reducing redundancy (http://revigo.irb.hr/). Circles represent GO terms and those clustered closer to each other represented similar GO terms. Disc colors (blue to yellow) represent de degree of GO enrichment (*p*-value) and disc size is proportional to the frequency of the GO term in the GO database (larger and smaller discs represent more general and more specific terms, respectively). **b** Heatmap showing distribution of RNAseq expression level (log10[FPKM+ 1], pale yellow to blue from less to more expressed) for DEGs belonging to the top enriched GO categories in *M. oryzae*-infected *MIR7695*-Ac plants (left panel). Heatmaps show upregulated (purple) and downregulated (green) DEGs (bifactorial analysis, middle panel; Monofactorial analysis for the given comparisons, right panel) Biological processes are indicated to the left. Data are means (*n* = 2). The full gene ID list is shown in Additional file 14: Table S10.

To further establish differences in the transcriptional response to pathogen infection between *MIR7695-Ac* and WT-Az plants, we used a two-factor analysis (genotype and treatment) of the full dataset of DEGs in each genotype. A total of 153 and 100 genes were identified as upregulated and downregulated, respectively, in *MIR7695*-Ac versus WT plants (Fig. 6a and Additional file 16: Figure S3d; Additional file 13: Table S9). AgriGO was used for GO enrichment analysis of DEGs in the bifactorial analysis, with clustering by Revigo (Fig. 6a). The GO terms over-represented in upregulated genes were clustered in the categories “response to stress” (biotic and oxidative stress), “response to stimulus”, and “secondary metabolism” (phenylpropanoids and terpenoids) (Fig. 6a, upper panel). Genes induced in *MIR7695-*Ac plants in the bifactorial analysis included defense-related genes, such as *PR* genes (*PR1*, *PR2*, *PR5* and *PR10* family members) and oxidative stress-related enzymes (e.g., several peroxidases) (Fig. 6b and Additional file 14: Table S10).

Phenylpropanoid biosynthetic genes were highly represented in the bifactorial analysis of DEGs. They included genes involved in the production of flavonoids (*CHS; F3H, FLS, DFR*) and monolignols, the building blocks of lignin (*CCR, CAD, COMT, PMT, LAC, PRX*) (Fig. 6b and Additional file 14: Table S10; additional information on phenylpropanoid and lignin biosynthesis genes that were differentially regulated in infected *MIR7695-*Ac plants is in Additional file 15: Figure S5a**)**. The expression of flavonoid biosynthesis genes is known to be induced by pathogen infection, and certain plant flavonoids exhibited antifungal activity [59]. The accumulation of lignin in secondary cell walls provides a physical barrier against pathogen invasion [60]. The expression of several peroxidases was upregulated in *MIR7695-Ac* versus WT-Az plants and also with pathogen infection. Peroxidases are key enzymes in the biosynthesis of lignin during resistance reactions via cross-linking of lignin monomers. A stronger induction of genes involved in flavonoid and lignin biosynthesis might play a role in protecting the *MIR7695-*Ac plants against *M. oryzae* infection. In addition, a important number of genes involved in the production of diterpenoid phytoalexins were among the top induced genes in *MIR7695*-Ac plants with infection (bifactorial DEGs) (Fig. 6b; Additional file 14: Table S10) as described below.

The GO term most represented in downregulated genes on bifactorial analysis of DEGs was “iron ion transport” (Fig. 6a, lower panel). This included genes related to Fe homeostasis, such *OsFRO2* (a Fe^3+^ reductase), *OsNAS2* (a nicotianamine synthase), *OsIRO2* TF, *OsHRZ1, OsHRZ2* ubiquitin ligases*, OsNRAMP1* (Fe^2+^ transporter), *OsMIR* (mitochondrial Fe-regulated gene), and *OsIROPT* (an oligopeptide transporter) (Fig. 6b; Additional file 14: Table S10).

**Fig. 7:**
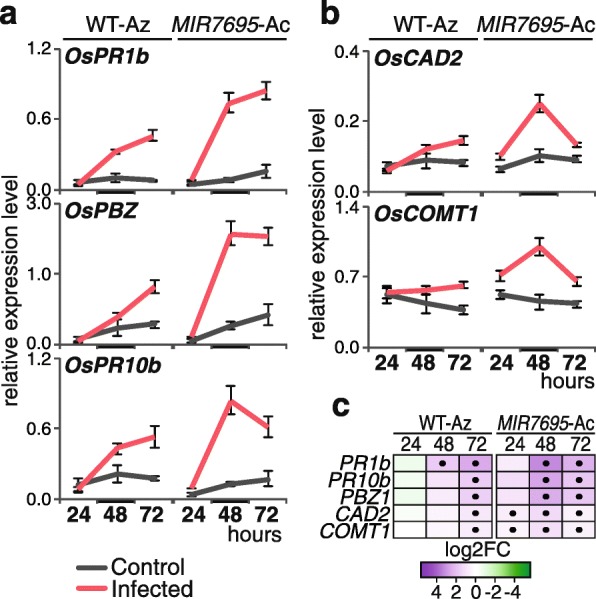
Expression of *PR* and lignin biosynthesis genes in WT-Az and *MIR7695*-Ac plants during blast infection. Plants were sprayed with a *M. oryzae* spore suspension. Leaves were collected at the indicated times (24, 48, 72 hpi). **a-b** Expression analysis of (**a**) *PR* (*OsPR1b, OsPBZ, OsPR10b*) and (**b**) lignin (*OsCAD2, OsCOMT1*) genes determined by RT-qPCR. Data are mean ± SE (*n* = 3; each sample consisted of a pool of 3 individual leaves). Mock-inoculated (control, grey) and *M. oryzae*-infected (red) plants. Time point used for RNAseq analysis (48 h) is labeled with a thick line in the x-axis. **c** Heatmap showing log2 FC for each transcript and each time (infected vs. control) as determined from RT-qPCR values (**a**-**b**). Upregulated (purple) and downregulated (green). Dots indicate significant differences (infected vs control) (Student *t* test, *p* < 0.05)

A more detailed expression analysis was performed for genes identified by bifactorial analysis, and their expression was examined by RT-qPCR at different times after inoculation with *M. oryzae* spores (24, 48 and 72 hpi). This analysis confirmed stronger induction of *PR* genes (*OsPR1b, OsPBZ, OsPR10b*) and lignin biosynthesis genes (*OsCAD2, OsCOMT1*) in *MIR7695-*Ac than WT-Az plants during *M. oryzae* infection (Fig. 7). Induction of *OsPBZ1* and other *OsPR10* family members is known to occur during *M. oryzae* infection and, when overexpressed, the genes confer pathogen resistance [61–63].

Altogether, comparative transcriptome analysis (bifactorial analysis) revealed stronger induction of defense-related genes in *MIR7695*-Ac (e.g., *PR*, oxidative stress-related, phenylpropanoid and diterpenoid phytoalexin biosynthesis genes), whereas genes that function in Fe homeostasis appear to be downregulated in *MIR7695-Ac* plants during *M. oryzae* infection.

### Regulation of Fe homeostasis-related genes in rice leaves during *M. oryzae* infection

In plant roots, two different mechanisms have been described for Fe uptake from the rhizosphere, the reducing and chelating strategies (strategies I and II, respectively) [64, 65]. Rice is unique in that as it uses both strategies. Besides strategy I and II genes, other genes contribute to Fe transport and/or mobilization through the plant. Although great progress has been made during the last years to identify mechanisms governing Fe uptake in roots, the regulation of Fe homeostasis genes in leaves is less understood.

We investigated the expression profile of Fe homeostasis-related genes in leaves of wild-type plants during *M. oryzae* infection. Genes examined were: *OsFRO2, OsIRO2, OsHRZ1, OsNRAMP1* and *OsIROPT1* (genes strongly downregulated in *MIR7695-*Ac plants). These genes were strongly upregulated early during infection (24–48 hpi) but downregulated at a later stage of the infection process (72 hpi) (Additional file 16: Figrue S6; WT-Az, infected vs mock). Upregulation of these genes early during infection of WT plants correlates with Fe accumulation at the sites of fungal penetration and infection sites, as revealed by histochemical analysis of *M. oryzae*-infected rice leaves (Fig. 1).

For a comparison, we examined the expression profile of Fe homeostasis genes in *MIR7695-*Ac plants. Four of the five genes examined were induced early during infection (24 hpi), as it was observed in WT plants, followed by a strong downregulation at 48 and 72 hpi (Additional file 16: Figure S6; *MIR7695-Ac*, infected vs mock). Therefore, downregulation of Fe homeostasis genes occurs earlier in *MIR7695-Ac* than WT-Az plants.

### Phytoalexins accumulate in *MIR7695-*ac plants during *M. oryzae* infection

**Fig. 8:**
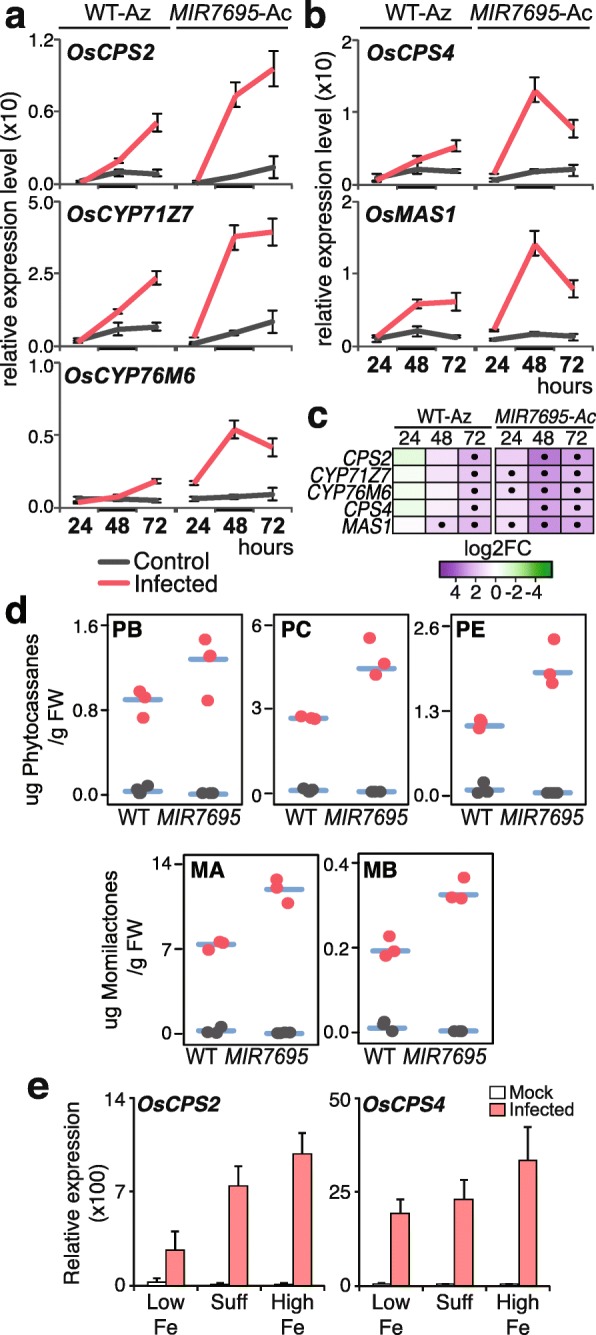
Expression of diterpenoid phytoalexin genes in WT-Az and *MIR7695*-Ac plants during blast infection. Plant material was treated as in Fig. 7. **a-b** RT-qPCR analysis of expression of (**a**) Phytocassane (*OsCPS2, OsCYP71Z7, OsCYP76M6*) and (**b**) momilactone (*OsCPS4, OsMAS1*) biosynthesis genes in rice leaves infected with *M. oryzae*. Data are mean ± SE (*n* = 3; each sample consisted of a pool of 3 individual leaves). Mock-inoculated (control, grey) and *M. oryzae*-infected (red) plants. **c** Heatmap showing log2 FC for each transcript and each time point (infected vs control) as determined from RT-qPCR values (**a**-**b**). Upregulated (purple) and downregulated (green). Dots indicate significant differences (infected vs control) (Student *t* test, *p* < 0.05). **d** Accumulation of diterpenoid phytoalexins, phytocassane E (PE), B (PB) and C (PC) (upper panels) and momilactone A (MA) and B (MB) (lower panels), in leaves of mock- and *M. oryzae*-infected plants. Each dot represents a biological replicate. FW, fresh weight. **e** RT-qPCR of expression of upstream diterpenoid biosynthetic genes (*OsCPS2* and *OsCPS4*) in mock- and *M. oryzae-*infected leaves of rice plants treated under three different Fe supply conditions (low, sufficient, high). Data are mean ± SE (*n* = 3), each sample consisting of a pool of 4 individual leaves)

Phytoalexins are low-molecular-weight antimicrobial compounds that accumulate in plant tissues during pathogen infection [66]. Major phytoalexins accumulating in rice leaves in response to *M. oryzae* infection are the diterpene phytoalexins momilactones, phytocasssenes and oryzalexins [67]. As previously mentioned, the expression of genes involved in the biosynthesis of diterpenoid phytoalexins, oryzalexins, phytocassenes and momilactones was induced to a higher level in *MIR7695-Ac* than WT-Az plants (at 48 hpi with *M. oryzae*) (see Fig. 6b**)**. For details on genes involved in diterpene phytoalexin biosynthesis with overexpression in *MIR7695* plants, see Additional file 15: Figure S5b. RT-qPCR analysis of diterpene phytoalexin biosynthesis genes at different times after inoculation with *M. oryzae* spores (24, 48, 72 hpi) confirmed earlier and stronger induction of these genes in *MIR7695-*Ac than WT-Az plants (Fig. 8a, b). Differences in pathogen-induced expression of these genes were more evident at 48 and 72 h after blast inoculation, as revealed by the higher fold change of gene expression (Fig. 8c).

To investigate whether superinduction of diterpenoid phytoalexin biosynthesis genes affects phytoalexin accumulation, we measured phytocassane and momilactone levels in leaves of *MIR7695*-Ac and WT-Az plants without and with infection. As expected, the expression of phytocassanes (B, C and E) and momilactones (A and B) was barely detected in non-infected rice leaves (Fig. 8d). Upon pathogen challenge, the accumulation of phytocassanes and momilactones increased in both WT-Az and *MIR7695-Ac* plants but was significantly higher in *MIR7695*-Ac than WT plants (Fig. 8d). These findings revealed that in response to pathogen infection, diterpenoid phytoalexin biosynthesis genes are induced earlier in *MIR7695-*Ac than WT-Az plants. *MIR7695-*Ac plants also accumulated higher levels of phytoalexins during pathogen infection. Knowing that diterpene phytoalexins have antifungal activity against *M. oryzae* [67–69], a higher *M. oryzae*-induced accumulation of phytoalexins in leaves of *MIR7695-Ac* plants might contribute to disease resistance in these plants.

Finally, we investigated whether Fe supply affects the expression of genes involved in the biosynthesis of diterpenoid phytoalexins in rice. We analyzed the effect of Fe supply (low, sufficient and high) on the expression of *OsCPS2* and *OsCPS4*, which function first cyclization steps in the phytoalexin biosynthetic pathway (Additional file 15: Figure S5b). The expression of these genes was barely detected in plants without infection (Fig. 8e). Upon pathogen challenge, the highest expression of phytoalexin genes occurred in plants grown under high Fe supply as compared with low or sufficient Fe (Fig. 8e), which supports that Fe supply affects phytoalexin biosynthesis. Presumably, a localized accumulation of Fe at the sites of pathogen penetration and/or invasion would activate the expression of phytoalexin biosynthetic genes for phytoalexin accumulation, thus arresting fungal colonization in infected leaves of *MIR7695-*Ac plants.

## Discussion

Although an increasing number of miRNAs have been shown to be differentially expressed in response to pathogen infection or nutrient stress, most of this research involved plants exposed to one or another type of stress separately. Furthermore, few studies aimed to understand the regulation of Fe homeostasis in rice during *M. oryzae* infection. Here, we present evidence of a miR7695-guided cleavage of *OsNramp6.8* transcripts encoding the NRAMP6 iron transporter from rice. Moreover, we investigated the role of miR7695 in the rice response to infection by *M. oryzae*. Upon challenge with *M. oryzae*, Fe accumulated near *M. oryzae* appressoria and in cells surrounding infected regions of rice leaf. Very recently, Dangol et al. reported that incompatible rice/*M.oryzae* interactions trigger iron- and ROS-dependent ferroptotic cell death in leaf sheaths of rice plants where iron accumulated at sites of infection to mediate the oxidative burst [22].. Activation-tagged *MIR7695* rice plants showed enhanced resistance and a stronger accumulation of iron at the sites of infection. On RNA-seq analysis, defense-related genes, including *PR* and diterpenoid biosynthetic genes were strongly induced along with blast resistance in *MIR7695-*Ac plants. Levels of phytoalexins during pathogen infection were higher in *MIR7695-*Ac than WT azygous plants and genes in the phytoalexin biosynthetic pathway were highly induced in rice plants grown under high Fe supply. This piece of evidence support that miR7695 positively regulates immune responses and establish links between defense signaling and Fe homeostasis in rice. However, the exact mechanisms by which Fe signaling regulates the expression of defense-related genes remains to be determined.

Being a foliar pathogen, *M. oryzae* has an absolute requirement for Fe from host tissues, so rice plants might capitalize on the toxicity or the essentiality of Fe to arrest *M. oryzae* invasion. Different scenarios can be considered. On the one hand, mechanisms that exploit Fe toxicity might be used by the host plant against *M. oryzae*. On the other, the host plant might develop withholding strategies to restrict Fe availability to the invading pathogen, a process that in humans and animals has been called “nutritional immunity” [70]. An examination of Fe distribution in *M. oryzae*-infected WT rice leaves revealed Fe accumulation in close vicinity of appressoria and in cells surrounding the infection sites, thus, reinforcing the notion that rice plants use strategies to locally increase Fe levels to prevent penetration and spread of the pathogen into the leaf tissue. Local accumulation of Fe would avoid Fe poisoning caused by a generalized accumulation of Fe in rice leaf while providing a signal for the activation of host immune responses. If so, this localized accumulation at the sites of pathogen penetration and invasion might mediate a localized oxidative burst that can be toxic to the invading pathogen. Local accumulation of H_2_O_2_ would also serve for cell-wall reinforcement (lignification, oxidative cross-linking of cell wall components) and induction of defense-related genes (e.g., *PR* genes).

Without infection, Fe preferentially accumulated in leaf stomata. In this respect, Fe has been shown to be important in regulating aperture of stomata [71]. During *M. oryzae* infection, a re-distribution of Fe appears to occur in the rice leaf, Fe moving around stomata and toward the sites of pathogen penetration and colonization. In support of this notion, a localized accumulation of Fe in cell wall appositions and subsequent defensive H_2_O_2_ production was previously linked to basal defense in wheat leaves after infection with *Blumeria graminis* f. sp. *tritici* [72]. Also, altered Fe distribution in Arabidopsis plants infected with the bacterial pathogen *Dickeya dadantii* was reported [73]. Although not proven, the activation of toxic oxidative bursts caused by localized accumulation of Fe in rice leaves might be important to restrict *M. oryzae* growth while maintaining normal plant development. Iron accumulation at the sites of pathogen infection was observed in both wild-type and *MIR7695-Ac* plants, the later ones accumulating more iron at the infection sites than wild-type plants.

During *M. oryzae* infection, genes involved in Fe homeostasis were strongly downregulated in leaves of *MIR7695-Ac* plants compared with WT plants. As previously mentioned, the rice plant uses a combined strategy for Fe uptake from the rhizosphere that has features of both strategy I (reduction of Fe^3+^ to Fe^2+^, a system that operates in roots of most non-graminaceous species) and strategy II (release of phytosiderophores by the root, typical of graminaceous species) [74]. Our results indicate that during *M. oryzae* infection, genes that function in Fe uptake via strategy I (e.g. *OsFRO2*) or strategy II (e.g. *OsIRO2*, *OsNAS2*) in roots are downregulated in leaves in both WT-Az and *MIR7695*-Ac plants. Other Fe homeostasis genes such as *NRAMP1* (a Fe transporter), *OsHRZ1* and *OsIROPT1* are also downregulated during infection. In line with this, the Fe homeostasis genes *TmFER1* and *TmNAS1* (marker genes for monitoring intracellular Fe status in wheat) were found downregulated in *B. graminis*-infected wheat leaves [72]. Furthermore, downregulation of Fe homeostasis genes was accompanied by cytosolic Fe depletion and induction of *PR* genes. A better understanding of the mechanisms involved in Fe homeostasis in rice leaf tissues is needed to know whether *M. oryzae* also provokes intracellular Fe depletion in rice leaves.

*MIR7695*-*Ac* plants exhibited resistance to *M. oryzae* infection, which is consistent with the phenotype of disease resistance observed in loss-of-function *OsNramp6* plants [48]. Disease resistance in *MIR7695-*Ac plants is associated with a basal expression of resistance genes and defense regulatory genes (e.g., *OsWRKY45*, *OsNAC4*) without pathogen infection and a superinduction of defense-related genes with infection. Thus, *MIR7695-*Ac plants mount a stronger defense response to pathogen infection, a response that is reminiscent of defense priming [75]. Whether defense responses are activated earlier in *MIR7695-*Ac than WT plants is unknown. Furthermore, proteins encoded by defense-related genes that are strongly induced during infection in *MIR7695-*Ac plants are known to possess antimicrobial activity (e.g., chitinases, β-1,3-glucanases, PR10 and LTP proteins), and their overexpression in plants confers pathogen resistance, including blast resistance [63, 76]. Stronger expression and induction of peroxidases is also a feature of *MIR7695-*Ac plants, these genes being typically induced in host plant tissues upon pathogen infection. Peroxidases are important for generating highly toxic environments by producing ROS species during resistance reactions [77] and for lignin biosynthesis (cross-linking of lignin monomers). A miR7695-mediated regulation of peroxidases might then function to generate an oxidative burst at the sites where Fe accumulates, thus helping to limit pathogen spread on the rice leaf. Also, an important number of genes involved in the flavonoid and lignin branches of the general phenylpropanoid pathway were upregulated in *MIR7695-Ac* versus WT plants (bifactorial analysis). The antifungal activity of phenylpropanoid compounds against phytopathogens has been reported [78–80]. The superactivation of these various defense genes might be responsible for the blast resistance phenotype observed in *MIR7695-*Ac plants.

Notably, upon pathogen challenge, diterpenoid phytoalexin biosynthesis genes were highly upregulated in *MIR7695-*Ac plants (bifactorial analysis), accompanied by increased accumulation of major rice phytoalexins. For some of these phytoalexins, antifungal activity against *M. oryzae* has been described [67–69]**.** Other studies proposed that rapid biosynthesis of diterpene phytoalexins contributes to resistance to *M. oryzae*, whereas delayed induction of these genes results in enhanced susceptibility to blast infection [67]. The accumulation of phytoalexins would enhance the ability to cope with pathogen infection in *MIR7695*-Ac plants.

## Conclusions

Overall, this study highlights the relevance of miR7695 in blast resistance via regulation of rice immune responses. Because miR7695 regulates *OsNramp6* encoding a Fe transporter from rice, these results support the existence of links between miR7695/*OsNramp6* functioning to control Fe signaling and defense signaling in rice. At the cellular level, *M. oryzae* infection altered Fe distribution in rice leaves, a process probably involving miR7695. Because miRNAs function as fine-tuners of gene expression instead of turning-on or turning-off target gene expression, miR7695 would be well suited to maintain appropriate Fe levels in host cells during pathogen infection. If so, miR7695 might well be involved in modulation of iron accumulation in tissues of the rice leaf which, in turn, would affect the expression of Fe homeostasis genes. The current challenge of basic and applied plant research is to understand interconnected regulations between miR7695-mediated mechanisms involved in Fe homeostasis and disease resistance in plants. Deciphering the mechanisms involved in Fe distribution and remobilization during *M. oryzae* infection with the participation of miR7695 will help in designing innovative strategies for blast disease control. Knowing how plants integrate immune responses and Fe signaling pathways is an issue of great importance in both basic and applied plant research.

## Methods

### Plant material, growth conditions and genotyping

Rice plants were grown at 28 °C with a 14 h/10 h light/dark cycle. The T-DNA insertion line for *MIR7695* (*O. sativa* cv. Tainung67, *japonica*) was obtained from the Taiwan Rice Insertion Mutant (TRIM) collection from the Academia Sinica of Taiwan ([49]; http://trim.sinica.edu.tw), and propagated under controlled conditions (CRAG greenhouse Service). For genotyping, genomic DNA was extracted as described [81] but with mixed alkyltri-methylammoniumbromide (MATAB) used as the extraction buffer (0.1 M Tris–HCl pH 8.0, 1.4 M NaCl, 20 mm EDTA, 2% MATAB, 1% PEG 6000, 0.5% sodium sulphite). PCR genotyping (100 ng DNA/PCR reaction) involved specific primers (*P1* and *P3*) and T-DNA–specific primers (*P2*) (Additional file 2: Table S1). T-DNA copy number was estimated as described [82].

For Fe treatment, 10 rice seeds were grown in 0.35-L pots containing soil (turface: vermiculite:quartz sand [2:1:3]) for 14 days and then watered with a half-strength Hoagland solution (5 mM KNO_3_, 5 mM Ca (NO_3_)_2_·4H_2_O, 2 mM MgSO_4_·7H_2_O, 1 mM NH_4_NO_3_, 0.5 mM KH_2_PO_4_ (pH to 6.0), 46.3 μM H_3_BO_3_, 9.1 μM MnCl_2_·4H_2_O, 0.76 μM ZnSO_4_·7H_2_O, 0.2 μM CuSO_4_·5H_2_O, 0.28 μM Na_2_MoO_4_·2H_2_O, 51.7 μM Fe-EDDHA). To assess the effect of Fe supply, the same nutrient solution was used but with a lower or higher Fe concentration (0.1 μM or 1 mM Fe-EDDHA). After 5 days of Fe treatment, plants were infected with *M. oryzae* spores (see below for inoculation method).

### Perls staining and DAB/H_2_O_2_ intensification

Rice leaves (mock- and blast-inoculated, 48 h post-infection [hpi]) were stained with Prussian blue dye according to [83] with some modifications. Briefly, rice leaves were vacuum-infiltrated in a fixing solution (chloroform:methanol:glacial acetic acid; 6:3:1, v/v) for 1 h and incubated overnight at room temperature. After washing with distilled water (three times), samples were vacuum-infiltrated with a pre-warmed (37 °C) staining solution (4% HCl and 4% K-ferrocyanide at equal volumes) for 1 h, incubated 1 h more at 37 °C in the same solution without vaccuum and washed three times with distilled water (Perls staining). For DAB intensification reaction, samples were incubated in a methanol solution (0.01 M NaN, 0.3% [v/v] H_2_O) for 1 h, washed with 0.1 M phosphate buffer pH 7.2, then incubated with the intensification solution (0.025% [w/v] DAB [Sigma], 0.005% [v/v] H_2_O in 0.1 M phosphate buffer, pH 7.2) for 15 min. The reaction was stopped by washing with distilled water. Leaves were mounted in glycerol 50% in glass slides and observed under a microscope (AixoPhot DP70 under with light).

### Chlorophyll content

The mean of 10 readings from the chlorophyll meter (SPAD 502 Plus Chlorophyll Meter, Spectrum Technologies) was obtained from the third leaf of rice plants grown in different Fe concentrations. The measurement was taken at the same position in all leaves.

### Blast resistance assays

The fungus *M. oryzae* (strain Guy-11, courtesy of Ane Sema) was grown in Complete Media Agar (CMA, 9 cm plates, containing 30 mg/L chloramphenicol) for 15 days at 28 °C under a 16 h/8 h light/dark photoperiod condition. *M. oryzae* spores were prepared as previously described [41]. Soil-grown plants (3–4 leaf stage) were infected by two different methods, 1) whole-plant spray inoculation assays [84], and 2) drop inoculation on detached leaves [85]. Briefly, the spray inoculation method consisted of spraying whole rice plants with a *M. oryzae* spore suspension (10^5^ spores/ml; 0.2 ml/plant) by using an aerograph at 2 atm of pressure. Plants were maintained overnight in the dark under high humidity. For the drop inoculation method, the second detached leaf was placed into square plate dishes (12 leaves/plate) with 1% (w/v) water agar containing kinetin (2 mg/l). Then, Whatman filter paper discs saturated with a *M. oryzae* spore suspension (10^4^–10^6^ spores/ml) were placed onto the upper face of the leaf for 60 h. The percentage of leaf area affected by blast lesions was determined at 4 days (drop-inoculated leaves) or 7 days (spray-inoculated leaves) post-inoculation with *M. oryzae* spores by using the APS Assess 2.0 program [86].

### Expression analysis

Total RNA was extracted from plant tissues by using TRizol reagent (Invitrogen). For northern blot analysis of rice miRNAs, RNAs were fractionated in a 17.5% denaturing polyacrylamide gel containing 8 M urea, transferred to nylon membranes and probed with a *γ* ^32^P-ATP end-labeled miR7695.3-3p oligonucleotide (Additional file 2: Table S1). Blots were pre-hybridized and hybridized in Perfect-Hyb Plus buffer (Sigma) at 42 °C. Hybridization signals were detected by using STORM Phosphorimager (GE Healthcare).

For quantitative RT-PCR (RT-qPCR), the first complementary DNA was synthesized from DNase-treated total RNA (1 μg) with High Capacity cDNA Reverse Transcription (Life technology, Applied Biosystems). Amplification involved 2 μl cDNA (5 ng/μl) in optical 96-well plates (Roche Light Cycler 480; Roche Diagnostics, Mannheim, Germany) with SYBR Green I dye and gene-specific primers (Additional file 2: Table S1). The *Ubiquitin1* gene (Os06g0681400) was used to normalize transcript levels.

### 5′-RLM-race

5′ RNA ligase-mediated rapid amplification of cDNA ends (5′ -RLM-RACE) was done using a GeneRacer™ kit according to the manufacturer’s instructions (Invitrogen, CA) but omitting the dephosphorylation and decapping steps. Briefly, 3 μg of DNAse-treated total RNA was ligated to a GeneRacer Oligo RNA Adapter. First-strand cDNA was synthesized using oligo-dT. Specific primers were used to amplify 5′ ends by nested PCR from cDNA (Additional file 2: Table S1). The nested PCR products were separated on a 2% agarose gel, gel purified, ligated to a Zero Blunt TOPO vector (Invitrogen, CA) transformed into Topo 10 cells and sequenced to determine the cleavage site in target genes. Specific control were done using the specific primers.

### RNA-seq library sample preparation and sequencing

Total RNA was extracted from rice leaves that had been treated or not with a *M. oryzae* spore solution following the whole-plant infection method (10^5^ spores/ml, 0.2 ml/plant, at 48 hpi) with the Maxwell 16 LEV Plant RNA Kit (Promega). Raw reads were checked for quality by using FastQC v0.11.3 (www.bioinformatics.babraham.ac.uk/projects/fastqc/) was used to check quality of raw reads; adapters were trimmed and removed with Trimmomatic v0.33 [87] (minimum quality score 35, minimum length 25). Reads obtained were mapped to the reference rice genome (MSU 7.0) provided with the reference gene annotation file (RGSP 7.0) by using STAR (v2.4.0j) [88]). Reads with mapping quality (MAPQ) < 30 were removed. FeatureCounts (v1.4.5-p1) [89] was used to perform read summarization at the gene level, with the strand-specific option “reversely stranded”. Statistical analysis of read counts was performed with R, with the HTSFilter package [90] to remove low-expressed genes and the edge R package [91] for differential expression analysis. To identify genes with significant difference in expression, a FDR cutoff < 0.05 and log2FC 1 ≤ or ≥ 1 was applied. Gene Ontology (GO) enrichment of differentially expressed genes involved Singular Enrichement analysis (SEA) using the AgriGO webtool (*p* < 0.01 Fisher’s test, TIGR genemodel) (http://bioinfo.cau.edu.cn/agriGO/) [92]. Enriched GO terms were grouped, summarized and 2D-plotted by semantic clustering with the online analysis tool ReviGO (http://revigo.irb.hr/) [93].

### Quantification of Rice Diterpene Phytoalexins

Leaf segments were collected from mock and *M. oryzae*-infected plants. Three biological replicates with two technical replicates each were performed. Approximately 200–300 mg of fresh plant material was soaked in 40 vol of 70% methanol and incubated at 4 °C overnight with constant rotation. A 1 ml aliquot was centrifuged at maximum speed to remove cell debris. Phytoalexins were quantified using 5 μl of the extract by LC-MS/MS as described [94]. Significant differences in phytoalexin accumulation were evaluated with ANOVA.

## Supplementary information

**Additional file 1: Figure S1.** Characterization and phenotype of *MIR7695*-Ac and wild-type azygous (WT-Az) plants.

**Additional file 2: Table S1.** Oligonucleotides used in this study

**Additional file 3: Table S2.** qPCR of T-DNA copy number in *MIR7695*-Ac mutant plants with the *sucrose phosphate synthase* (*SPS*) gene as an endogenous reference.

**Additional file 4: Figure S2.**Resistance of *MIR7695*-Ac mutant plants to *M. oryzae* infection.

**Additional file 5: Figure S3.** Differentially expressed genes (DEGs) in leaves of *MIR7695*-Ac mutant plants relative to WT-Az plants, under non-infection or infection.

**Additional file 6: Table S3.** Differentially expressed genes (DEGs) in *MIR7695*-Ac plants relative to wild-type azygous (WT-Az) plants (*MIR7695*-Ac vs WT-Az).

**Additional file 7: Table S4.** Gene Ontology terms enriched in *MIR7695*-Ac versus WT-Az plants according to molecular function.

**Additional file 8: Table S5.** DEGs in *MIR7695*-Ac versus WT-Az plants.

**Additional file 9: Figure S4.** Validation of RNAseq data by qRT-PCR.

**Additional file 10: Table S6.** DEGs in WT plants after infection with *M. oryzae* (48 h post-infection [hpi]) (INF-WT-Az vs. WT-Az).

**Additional file 11: Table S7.** DEGs in *MIR7695*-Ac plants after infection with *M. oryzae* (48 hpi) (INF-*MIR7695*-Ac vs. *MIR7695*-Ac).

**Additional file 12: Table S8.** *Pathogenesis-related* (*PR*) gene expression in WT-Az and *MIR7695*-Ac plants during *M. oryzae* infection at 48 hpi.

**Additional file 13: Table S9**. DEGs in bifactorial analysis.

**Additional file 14: Table S10**. DEGs in the top GO categories that are overrepresented in *MIR7695*-Ac with *M. oryzae* infection (bifactorial analysis).

**Additional file 15: Figure S5.** Pathways for the biosynthesis of phenylpropanoids and diterpenoid phytoalexins in rice.

**Additional file 16: Figure S6.** RT-qPCR analysis of expression pattern of Fe homeostasis genes in WT-Az and *MIR7695*-Ac plants with *M. oryzae* infection.

## Data Availability

The RNA sequence datasets generated during the current study will be available after May 2020 at the National Center for Biotechnology Information (NCBI) Gene Expression Omnibus (GEO) with the GSE122258 accession number (https://www.ncbi.nlm.nih.gov/geo/query/acc.cgi?acc=GSE122258). Until this date, the datasets are available from the corresponding author on reasonable request.

## Abbreviations

DEG: Differentially Expressed Gene
Fe: Iron
GO: Gene Ontology
LTP: Lipid Transfer protein
miR: MicroRNA
Nramp6: Natural Resistance-Associated Macrophage Pathogen 6
PR: Pathogenesis-Related
R: Resistance
ROS: Reactive Oxygen Species
TF: Transcription Factor
