# Supplementary material for: Osa-miR7695 enhances transcriptional priming in defense responses against the rice blast fungus
Source: BMC Plant Biol. 2019 Dec 18;19:563. doi: 10.1186/s12870-019-2156-5 (PMC6921540; doi:10.1186/s12870-019-2156-5)
Supplement: Supplementary file 1 — Additional file 1: Figure S1. Characterization and phenotype of MIR7695-Ac and wild-type azygous (WT-Az) plants. [file 12870_2019_2156_MOESM1_ESM.pdf]

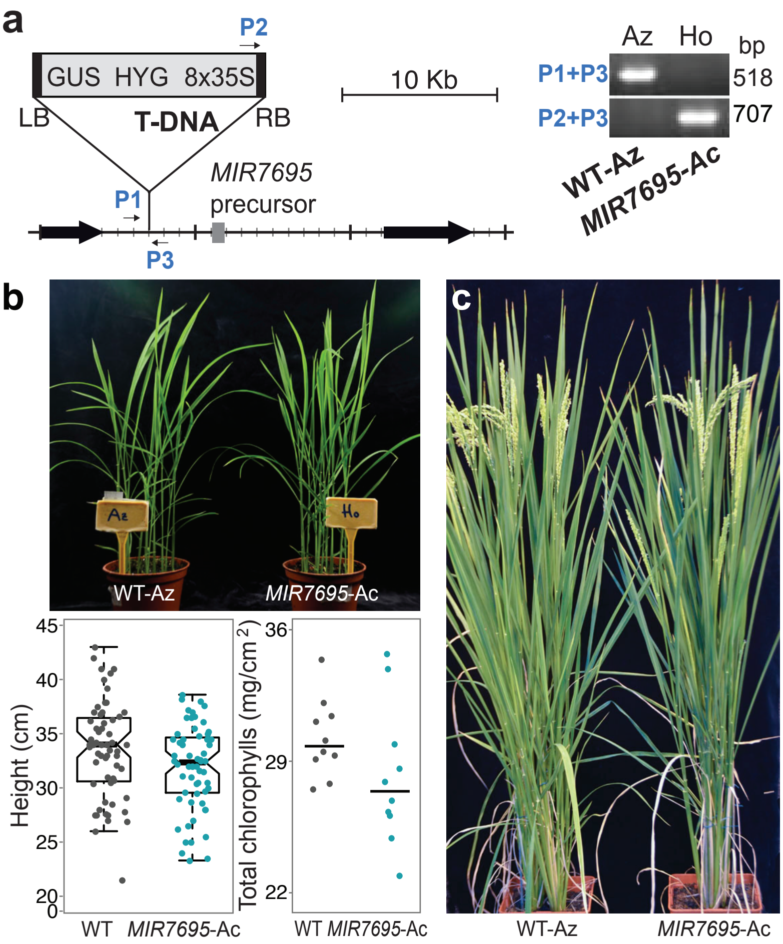

Figure S1. Characterization and Phenotype of *MIR7695*-Ac and wild-type azygous (WT-Az) plants.

(a) Schematic representation of the T-DNA insertion mutant from the TRIM collection (M0107013). GUS, β-glucuronidase; HYG, hygromycin phosphotransferase; 8x35S, Cauliflower mosaic virus 35S enhancer; RB, Right border; LB, Left border. (left panel) PCR genotyping of mutant plants with specific primers (P1, P2, P3; Additional file 2: Table S1) (right panel). Ho, homozygous for the T-DNA insertion; Az, segregated azygous.

(b) Soil-grown *MIR7695*-Ac and WT-Az plants were grown under greenhouse conditions for 3 weeks(s) and under a 14 h/10 h light/dark cycle, at 26 ± 2ºC. Height and chlorophyll content of *MIR7695*-Ac and WT-Az plants (left and right panels, respectively). Box plots show median, quartiles (boxes) and range (whiskers) for plant height measurements (n = 60). Notches indicate the 95% confidence interval of the median. Differences between *MIR7695*-Ac and WT-Az plants were not statistically significant.

(c) *MIR7695*-Ac and WT-Az plants at the maturity stage. Plants were grown for 3 months as in (a)
