# Supplementary material for: Osa-miR7695 enhances transcriptional priming in defense responses against the rice blast fungus
Source: BMC Plant Biol. 2019 Dec 18;19:563. doi: 10.1186/s12870-019-2156-5 (PMC6921540; doi:10.1186/s12870-019-2156-5)
Supplement: Supplementary file 4 — Additional file 4: Figure S2.Resistance of MIR7695-Ac mutant plants to M. oryzae infection. [file 12870_2019_2156_MOESM4_ESM.pdf]

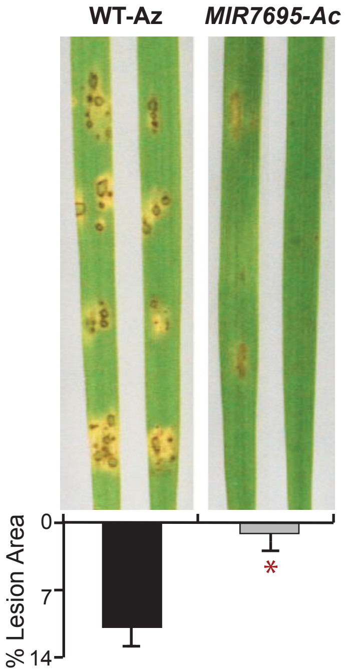

Figure S2. Resistance of *MIR7695*-Ac mutant plants to *M. oryzae* infection.
Disease resistance was determined by local inoculation of detached leaves with *M. oryzae* spore suspension at 10E5 spores/ml. Leaves were photographed at 4 days post-inoculation. Panel below, percentage of leaf area affected by blast lesions as determined by image analysis (APS Assess 2.0) (Lamari 2008). Data are mean ±SE (n=7). Three independent infection assays were performed with similar results. *P<0.05
