# Supplementary material for: Osa-miR7695 enhances transcriptional priming in defense responses against the rice blast fungus
Source: BMC Plant Biol. 2019 Dec 18;19:563. doi: 10.1186/s12870-019-2156-5 (PMC6921540; doi:10.1186/s12870-019-2156-5)
Supplement: Supplementary file 5 — Additional file 5: Figure S3. Differentially expressed genes (DEGs) in leaves of MIR7695-Ac mutant plants relative to WT-Az plants, under non-infection or infection. [file 12870_2019_2156_MOESM5_ESM.pdf]

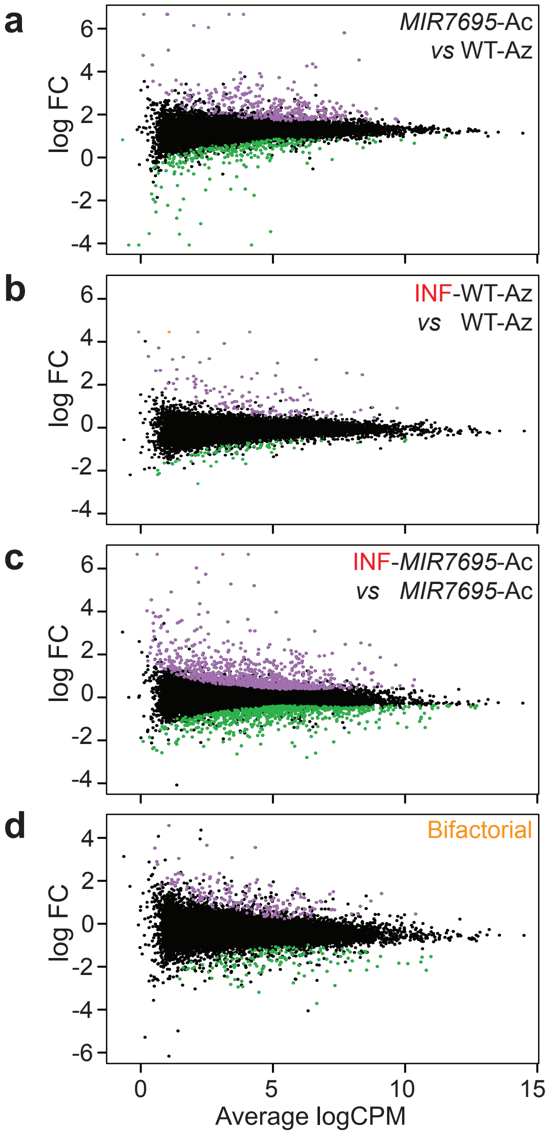

Figure S3. Differentially expressed genes (DEGs) in leaves of *MIR7695*-Ac mutant plants relative to WT-Az plants, under non-infection or infection.
Leaves of 3-week-old rice plants were mock-inoculated (WT-Az, *MIR7695*-Ac) or inoculated with *M. oryzae* spores (INF-WT-Az, INF-*MIR7695*-Ac). Leaves were collected at 48 h post-inoculation. MA Plots of logarithmic fold changes (LogFCs) to average count size in RNA-seq analysis of *MIR7695*-Ac plants vs WT-Az plants across different conditions. DEGs are highlighted in purple (upregulated) and green (downregulated) ($p < 0.05$, FDR < 0.05, n=2).
