# Supplementary material for: Osa-miR7695 enhances transcriptional priming in defense responses against the rice blast fungus
Source: BMC Plant Biol. 2019 Dec 18;19:563. doi: 10.1186/s12870-019-2156-5 (PMC6921540; doi:10.1186/s12870-019-2156-5)
Supplement: Supplementary file 9 — Additional file 9: Figure S4. Validation of RNAseq data by qRT-PCR. [file 12870_2019_2156_MOESM9_ESM.pdf]

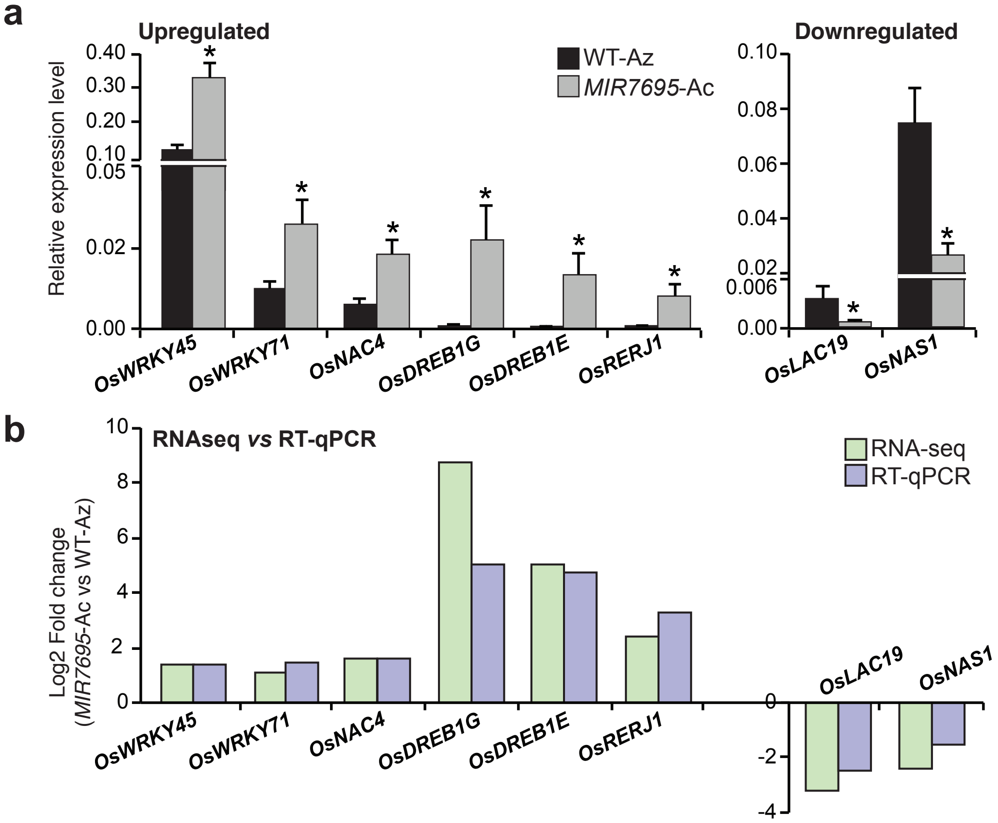

Figure S4. Validation of RNAseq data by qRT-PCR.

Leaves of 3-week-old WT-Az and *MIR7695*-Ac plants were used for RT-qPCR analysis (n=3).

(a) Expression of selected genes upregulated (left panel) or downregulated (right panel) in *MIR7695*-Ac vs WT-Az by RNA-seq. Data are mean ± SE (n=3) and were normalized to the rice *Ubiquitin* (Os06g0681400). Gene-specific primers are in Additional file 2: Table S1 *P<0.05

(b) Comparison of RNA-seq and RT-qPCR fold change values obtained for DEGs in (a).
