# Supplementary material for: Osa-miR7695 enhances transcriptional priming in defense responses against the rice blast fungus
Source: BMC Plant Biol. 2019 Dec 18;19:563. doi: 10.1186/s12870-019-2156-5 (PMC6921540; doi:10.1186/s12870-019-2156-5)
Supplement: Supplementary file 15 — Additional file 15: Figure S5. Pathways for the biosynthesis of phenylpropanoids and diterpenoid phytoalexins in rice. [file 12870_2019_2156_MOESM15_ESM.pdf]

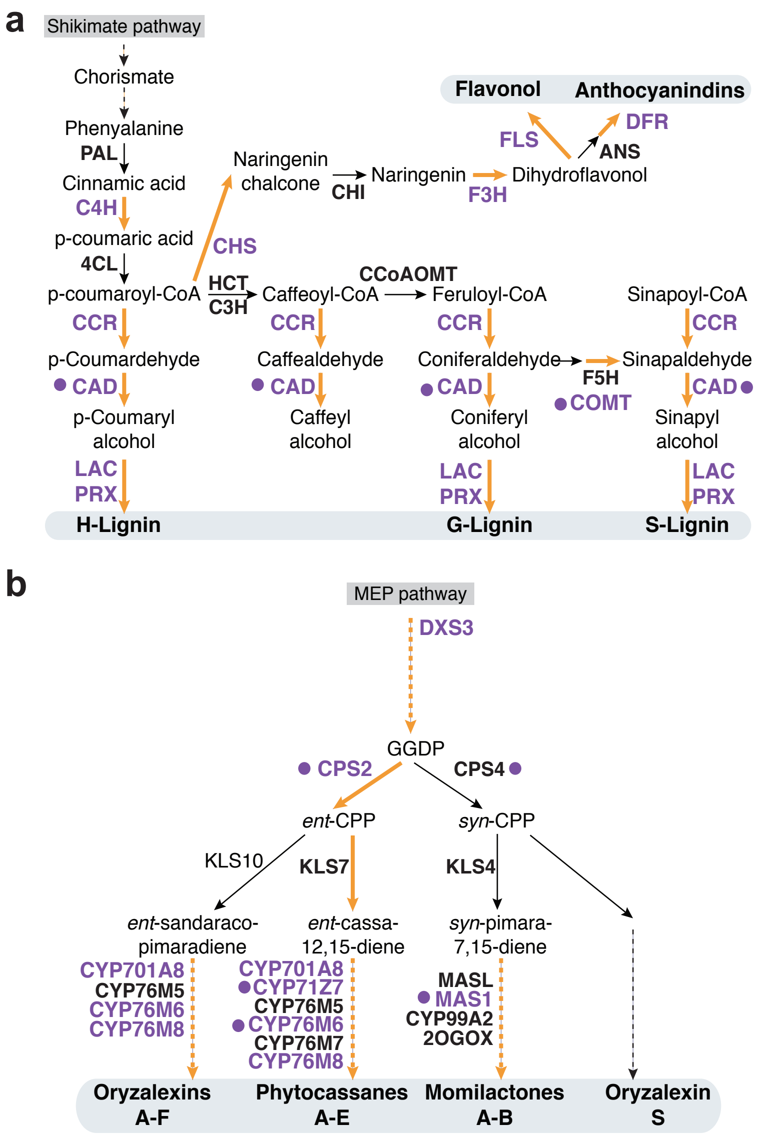

Figure S5. Pathways for the biosynthesis of phenylpropanoids (a) and diterpenoid phytoalexins (b) in rice. RNA-seq analysis of genes upregulated in *MIR7695*-Ac versus WT-Az plants during *M. oryzae* infection (in purple; log2FC ≥ 1; pval <0.05; FDR <0.05).

(a) Simplified scheme of the monolignol and flavonoid branches of the phenylpropanoid metabolism pathway. PAL, phenylalanine ammonia lyase; C4H, cinnamate-4-hydroxylase; 4CL, 4-coumaroyl-CoA ligase; CHS, chalcone synthase; CHI, chalcone isomerase; F3H, flavanone 3-hydroxylase; FLS, flavonol synthese; ANS, Anthocyanidin synthese; DFR, dihydroflavonol reductase; HCT, hydroxycinnamoyl transferase; C3H, coumarate 3-hydroxylase; CCoAOMT, caffeoyl/CoA-3-O-metheltransferase; CCR, cinnamoyl-CoA reductase; CAD, cinnamyl alcohol dehydrogenase; F5H, ferulate 5-hydroxylase; COMT, caffeic acid 3-Omethyltransferase; LAC, laccase; PRX, peroxidase. RT-qPCR of upregulated genes (purple dot) (see Fig. 7).

(b) Diterpenoid phytoalexin biosynthesis pathway. CPS, ent-Copalyl diphosphate synthase; KSL, kaurene synthase-like; MAS, momilactone A synthase, CYP, Cytochrome P450. RT-qPCR of upregulated genes (purple dot) (see Fig. 8).
