# Supplementary material for: Osa-miR7695 enhances transcriptional priming in defense responses against the rice blast fungus
Source: BMC Plant Biol. 2019 Dec 18;19:563. doi: 10.1186/s12870-019-2156-5 (PMC6921540; doi:10.1186/s12870-019-2156-5)
Supplement: Supplementary file 16 — Additional file 16: Figure S6. RT-qPCR analysis of expression pattern of Fe homeostasis genes in WT-Az and MIR7695-Ac plants with M. oryzae infection. [file 12870_2019_2156_MOESM16_ESM.pdf]

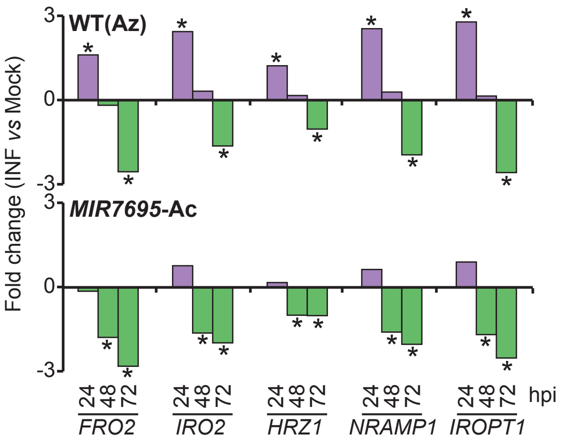

Figure S6. RT-qPCR analysis of expression pattern of Fe homeostasis genes (*OsFRO2, OsIRO2, OsHRZ1, OsNRAMP1, OsIROPT1*) in WT-Az and *MIR7695*-Ac plants with *M. oryzae* infection. The plant material was treated as in Fig. 7. Data are mean±SE (n=3) after normalization to Ubiquitin expression. *$P<0.05$
